# Identification of *LsPIN1* gene and its potential functions in rhizome turning of *Leymus secalinus*

**DOI:** 10.1186/s12864-022-08979-7

**Published:** 2022-11-16

**Authors:** Jialin Li, Hongmei Li, Ning Yin, Xiaoyan Quan, Wenbo Wang, Qiuli Shan, Siqi Wang, Ramon Santos Bermudez, Wenxing He

**Affiliations:** School of Biological Science and Technology, University of Jinan, Jinan, 250022 China

**Keywords:** Auxin transport, *Leymus secalinus*, *LsPIN1*, Gravity response, Rhizome

## Abstract

**Background:**

Continuous tilling and the lateral growth of rhizomes confer rhizomatous grasses with the unique ability to laterally expand, migrate and resist disturbances. They play key roles especially in degraded grasslands, deserts, sand dunes, and other fragile ecological system. The rhizomatous plant *Leymus secalinus* has both rhizome buds and tiller buds that grow horizontally and upward at the ends of rhizome differentiation and elongation, respectively. The mechanisms of rhizome formation and differentiation in *L. secalinus* have not yet been clarified.

**Results:**

In this study, we found that the content of gibberellin A3 (GA_3_) and indole-3-acetic acid (IAA) were significantly higher in upward rhizome tips than in horizontal rhizome tips; by contrast, the content of methyl jasmonate and brassinolide were significantly higher in horizontal rhizome tips than in upward rhizome tips. GA_3_ and IAA could stimulate the formation and turning of rhizomes. An auxin efflux carrier gene, *LsPIN1*, was identified from *L. secalinus* based on previous transcriptome data. The conserved domains of *LsPIN1* and the relationship of *LsPIN1* with *PIN1* genes from other plants were analyzed. Subcellular localization analysis revealed that LsPIN1 was localized to the plasma membrane. The length of the primary roots (PRs) and the number of lateral roots (LRs) were higher in *Arabidopsis thaliana* plants overexpressing *LsPIN1* than in wild-type (Col-0) plants. Auxin transport was altered and the gravitropic response and phototropic response were stronger in *35S:LsPIN1* transgenic plants compared with Col-0 plants. It also promoted auxin accumulation in root tips.

**Conclusion:**

Our findings indicated that *LsPIN1* plays key roles in auxin transport and root development. Generally, our results provide new insights into the regulatory mechanisms underlying rhizome development in *L. secalinus*.

**Supplementary Information:**

The online version contains supplementary material available at 10.1186/s12864-022-08979-7.

## Background

Rhizomes are one of the forms of modified stems that grow horizontally underground [1]. They serve as both vegetative and propagative organs in clonal plants; the ability of rhizomes to permit asexual propagation likely explains their presence in several perennial grasses and monocot plants [2–4]. One characteristic of rhizome development is that new buds on rhizomes can either grow underground to form new rhizomes or grow vertically to form new clonal ramet, which are often referred to as tillers [4–8]. The asexual reproductive process known as tillering is unique to monocotyledons. Several herbaceous plants possess underground rhizomes, and these are formed through continuous tillering, which can generate a network of underground rhizomes and their corresponding aboveground ramet [1, 9]. The continuous differentiation and lateral extension of rhizomes confer rhizomatous grasses with strong abilities of horizontal expansion, migration and anti-disturbance; they are also morphologically plastic, which allows them to respond to changes in the surrounding environment and even select habitats [1, 9–12]. When growth conditions are unfavorable, rhizomes can survive underground; however, when growth conditions become favorable, tiller buds can emerge rapidly. In recent years, some studies have examined tiller phenotypes and the genetic regulatory mechanisms in various rhizomatous species such as *oryza longistaminata*, tropical lotus, sorghum, bamboo, and ginger [1, 2, 4, 6, 13].

In rhizomatous plants, there are both rhizome buds and tiller buds; both bud types originate from axillary buds, and they can differentiate into either spreading or clumping ramets [9, 14]. Compared to our understanding of the molecular mechanisms underlying the characteristics of axillary bud and determinations of growth, little is known about the mechanisms underlying the development of rhizome buds and tiller buds. Furthermore, the regulatory processes controlling the development of rhizome buds and tiller buds in single plants remain unclear. Various internal factors and external stimuli regulate the differentiation and development of rhizomes.

Plant hormones are important regulators involved in growth and development of plants; they often act as chemical signals, and their effects on target genes are mediated via several signal transduction pathways [15–18]. Rhizome development is a special development process, and many studies have examined the roles of plant hormones in rhizome development. In a rhizomatous genotype of tall fescue plants, 6-benzylaminopurine (BAP) and gibberellin A3 (GA_3_) could promote rhizome formation and elongation, respectively [19]. The content of indole-3-acetic acid (IAA), zeatin riboside (ZR), and gibberellin A4 (GA_4_) increased in new rhizomes regenerated from rhizome nodes, but ABA content was decreased [7]. Concentrations of IAA, zeatin, and gibberellic acid in rhizome buds were high prior to the formation of bamboo shoots [20]. In vitro induction of rhizome in *Geodorum densiflorum*, α-naphthalene acetic acid (NAA) and 6-BA significantly increased the rate of rhizome formation [21]. Several hormone-related genes and transcription factors involved in rhizome development have been identified in some rhizomatous plants [6, 22, 23]. For example, *adventitious rootless1* (*ARL1*) regulates the gravity orientation of roots and tillers in rice by regulating or responding to the polar distribution and transport of auxin [24]. The *GRAS* gene family member *MONOCULM1* (*MOC1*) regulates the axillary bud initiation and tiller outgrowth in rice [5]. OsTB1 negatively regulates tiller outgrowth in rice [25]. AtPIN1 (auxin efflux carriers PIN-FORMED1) is involved in lateral root (LR) organogenesis, morphogenesis, photoresponse and auxin transport [26–28]. Auxin and strigolactone play joint roles in regulating shoot branching, and *AtPIN1* is particularly important in this process [29]. *MdPIN1* overexpression affected auxin transport and root development and promoted phototropism and geotropism in *Arabidopsis thaliana* [30]. The overexpression or silencing of *OsPIN1* using transgenic methods can affect auxin-dependent adventitious root emergence and tillering, indicating that OsPIN1 plays a key role in root formation and tillering [31]. These studies have greatly enhanced our understanding of the molecular mechanisms underlying rhizome development.

The perennial and rhizomatous grass *Leymus secalinu* has both rhizome buds and tiller buds that grow horizontally and upward at the ends of rhizome differentiation and elongation, respectively. Rhizomes can be lengthened through growth of the tips of horizontal rhizomes, and new tillers can be formed via growth of the tip of upward rhizomes. Continuous tilling, the lateral growth of rhizomes, and the growth of adventitious roots result in the formation of a complex network that confers *L. secalinus* with resistance to sand storms and the ability to stabilize sand land [32, 33]. As a typical clonal plant, *L. secalinus* plays key ecological roles in degraded grasslands, deserts, sand dunes, and other fragile ecosystems [33]. Although several studies have examined the ecological adaptation and physiological integration of rhizomes in *L. secalinus*, no studies have examined the mechanism underlying the formation of rhizome buds and tiller buds in this species. Identifying potential genes that play important roles in the formation and development of rhizome turn can aid the development of approaches for promoting rapid canopy establishment.

In this study, we characterized differences in the roles of endogenous hormones in regulating the development of horizontal and upward rhizomes. We identified the *LsPIN1* gene, which was highly similar to *AtPIN1*, and found that it was significantly differentially expressed in horizontal and upward rhizomes of *L. secalinus*. Overexpression of *LsPIN1* in *A. thaliana* revealed that *LsPIN1* played an important role in auxin transport and root development, which was involved in gravitropic response and phototropism. The results of this study shed new light on the molecular mechanisms underlying the formation and development of rhizomes in *L. secalinus*.

## Results

### Rhizome phenotypes and the content of endogenous hormones in horizontal and upward rhizome tips of *L. secalinus*

**Fig. 1:**
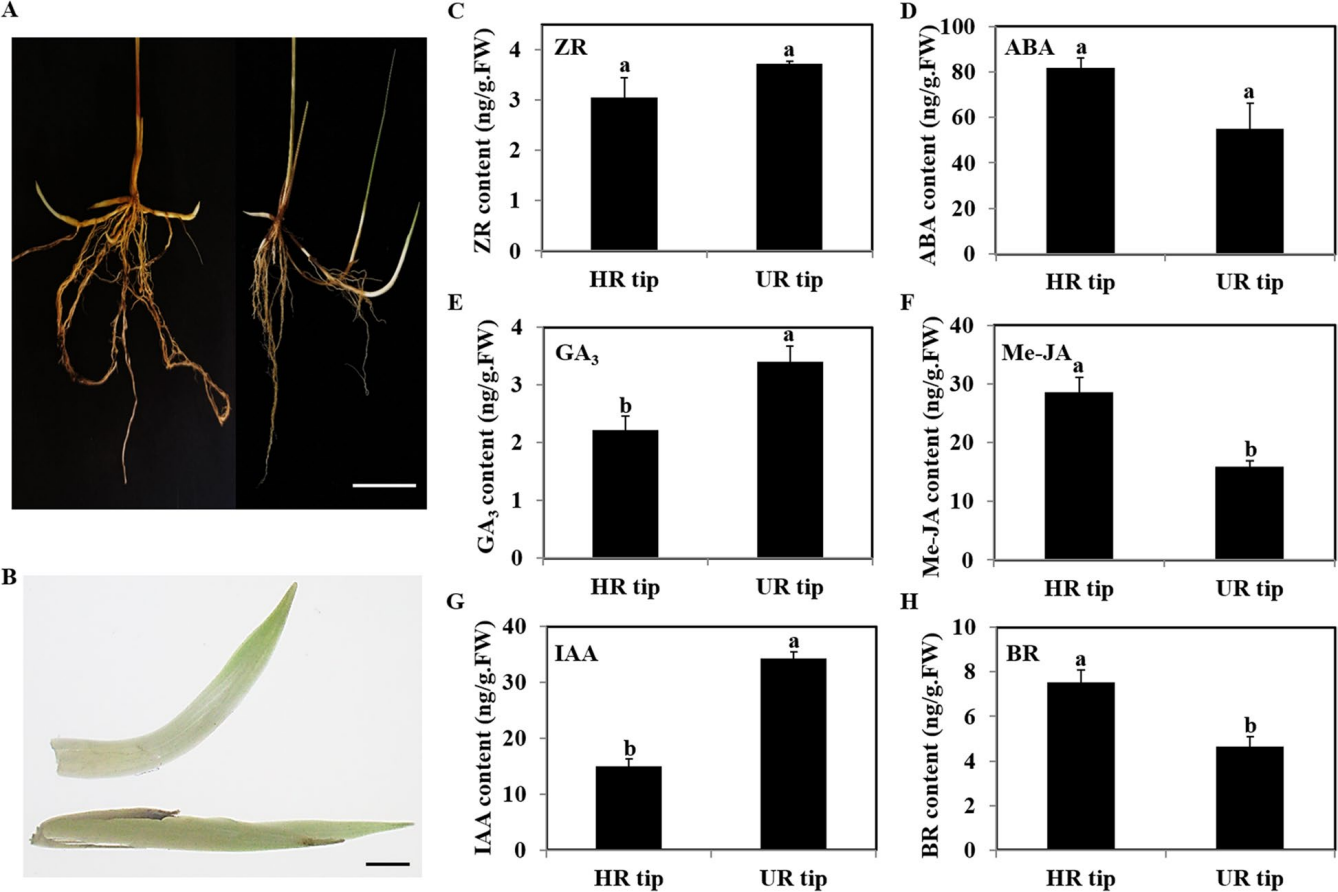
Phenotypes of *L. secalinus* rhizomes and the content of endogenous hormones in horizontal and upward rhizome tips. **a** Phenotypes of rhizomes grown in nutrient sandy soil after 20 d in an incubator. **b** The horizontal rhizome tip (HR tip) and upward rhizome tip (UR tip). The content of endogenous hormones in the two types of rhizome tips: (**c**) zeatin riboside (ZR); (**d**) abscisic acid (ABA); (**e**) gibberellin A3 (GA_3_); (**f**) methyl jasmonate (Me-JA); (**g**) indole-3-acetic acid (IAA); and (**h**) brassinolide (BR). Error bars were the standard errors (SE). Different lowercase letters represented significant differences (*P* < 0.05). Scale bars in A and B were 1 cm

The rhizome phenotypes of 20-d-old *L. secalinus* seedlings were shown in Fig. 1a. Each seedling contained two types of rhizome tips that grew horizontally and upward at the ends of rhizome differentiation and elongation, respectively (Fig. 1a-b). The horizontal rhizome tips lengthened the growth of the rhizome, whereas the upward rhizome tips lead to the formation of new tillers. The content of six endogenous hormones, ZR, ABA, GA_3_, Me-JA, IAA, and brassinolide (BR) in horizontal and upward rhizome tips was determined (Fig. 1c-h). The content of GA_3_ and IAA was significantly higher in upward rhizome tips than in horizontal rhizome tips; by contrast, the content of Me-JA and BR was higher in horizontal rhizome tips than in upward rhizome tips (Fig. 1e-h). These findings suggested that GA_3_, IAA, Me-JA, and BR might be involved in rhizome turning. However, no significant difference in the ZR and ABA content was observed between horizontal and upward rhizome tips, indicating that the two hormones might have no effect on the turning of the rhizome tips in *L. secalinus* (Fig. 1c-d).

### Effects of IAA and GA_3_ on rhizome formation and turning

**Fig. 2:**
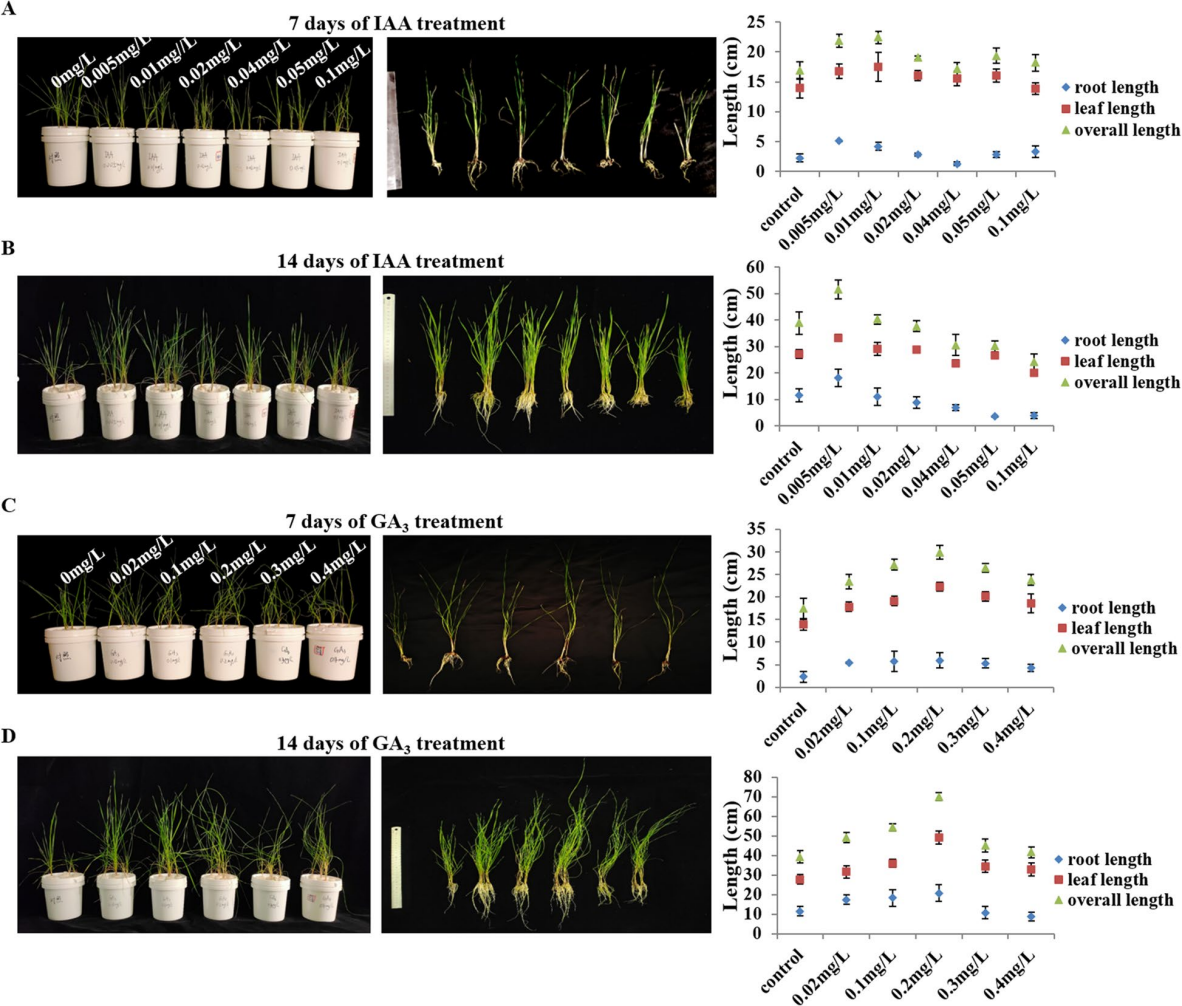
The growth of *L. secalinus* following treatment with different GA_3_ and IAA concentrations in a hydroponic system. **a** *L. secalinus* phenotypes at 7 d following treatment with 0.005 mg/L, 0.01 mg/L, 0.02 mg/L, 0.04 mg/L, 0.05 mg/L, and 0.1 mg/L IAA; the root length, leaf length and overall length of *L. secalinus* were measured. **b** *L. secalinus* phenotypes at 14 d following the IAA treatments in (**a**). **c** *L. secalinus* phenotypes at 7 d following treatment with 0 mg/mL (CK), 0.02 mg/L, 0.1 mg/L, 0.2 mg/L, 0.3 mg/L, and 0.4 mg/L GA_3_; the root length, leaf length, and overall length of *L. secalinus* were measured. **d** *L. secalinus* phenotypes at 14 d following the GA_3_ treatments in (**c**). Error bars indicate SE calculated from the results of three independent experiments

**Fig. 3:**
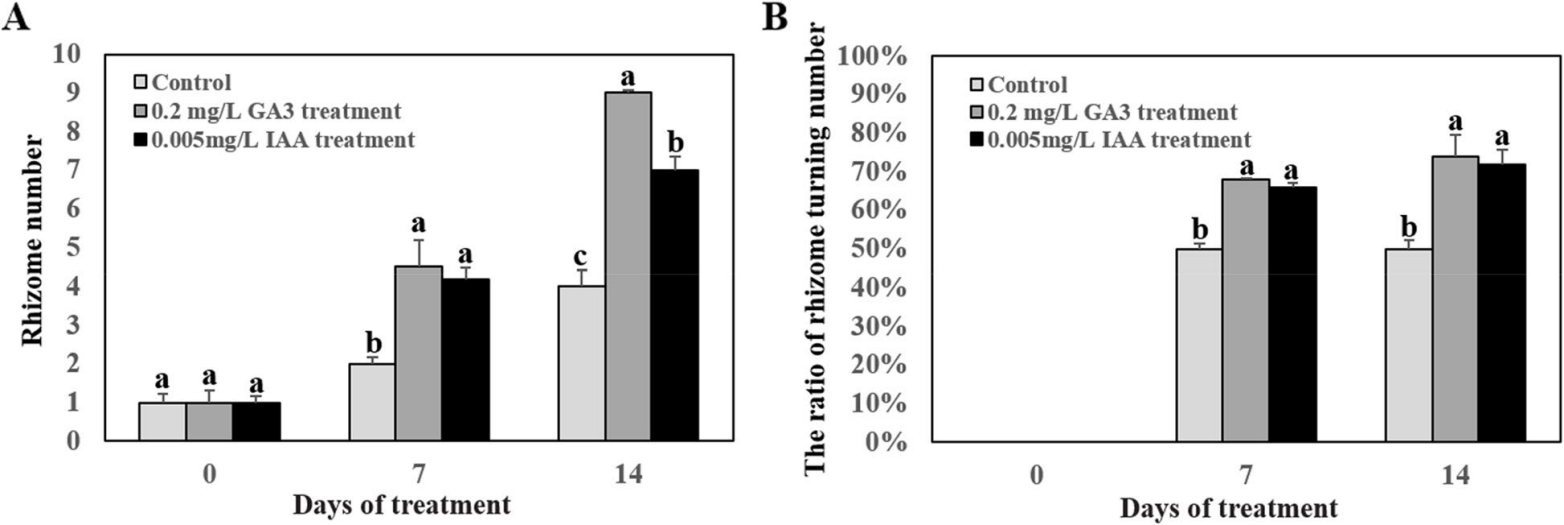
Effects of GA_3_ and IAA on *L. secalinus* rhizomes. **a** Average number of rhizomes per plant following treatment with 0.2 mg/L GA_3_ and 0.005 mg/L IAA at various time points. **b** The ratio of rhizome turning number to total rhizome number following treatment with 0.2 mg/L GA_3_ and 0.005 mg/L IAA at various time points. Error bars indicated SE calculated from the results of three independent experiments. Different lowercase letters represented significant differences (*P* < 0.05)

Previous studies have shown that auxin and GA_3_ play important roles in the growth and development of rhizomes or roots [15, 34, 35]. The above studies have revealed significant differences in the content of IAA and GA_3_ in horizontal and upward rhizome tips of *L. secalinus*, suggesting that IAA and GA_3_ might affect rhizome formation and turning. To clarify the effects of IAA and GA_3_ on rhizome initiation and turning, *L. secalinus* seedlings were grown in hydroponic nutrient solution with different concentrations of IAA and GA_3_. Following 14 d of treatment, the number of rhizomes per plant, the ratio of rhizome turning per plant, and the length of the roots were significantly higher in IAA and GA_3_-treated plants compared with control plants (Fig. 2, Fig. 3). The number of rhizomes per plant increased from 1 to 9 and the ratio of rhizome turning number to total rhizome number increased from 50 to 74% in the 0.2 mg/L GA_3_ treatment. The number of rhizomes per plant increased from 1 to 7 and the ratio of rhizome turning number to total rhizome number increased from 50 to 72% in the 0.005 mg/L IAA treatment (Fig. 3). Overall, these findings indicated that the application of appropriate concentrations of IAA and GA_3_ could promote the formation and turning of rhizomes in *L. secalinus*.

### Identification and analysis of *LsPIN1* related to rhizome development in *L. secalinus*

**Fig. 4:**
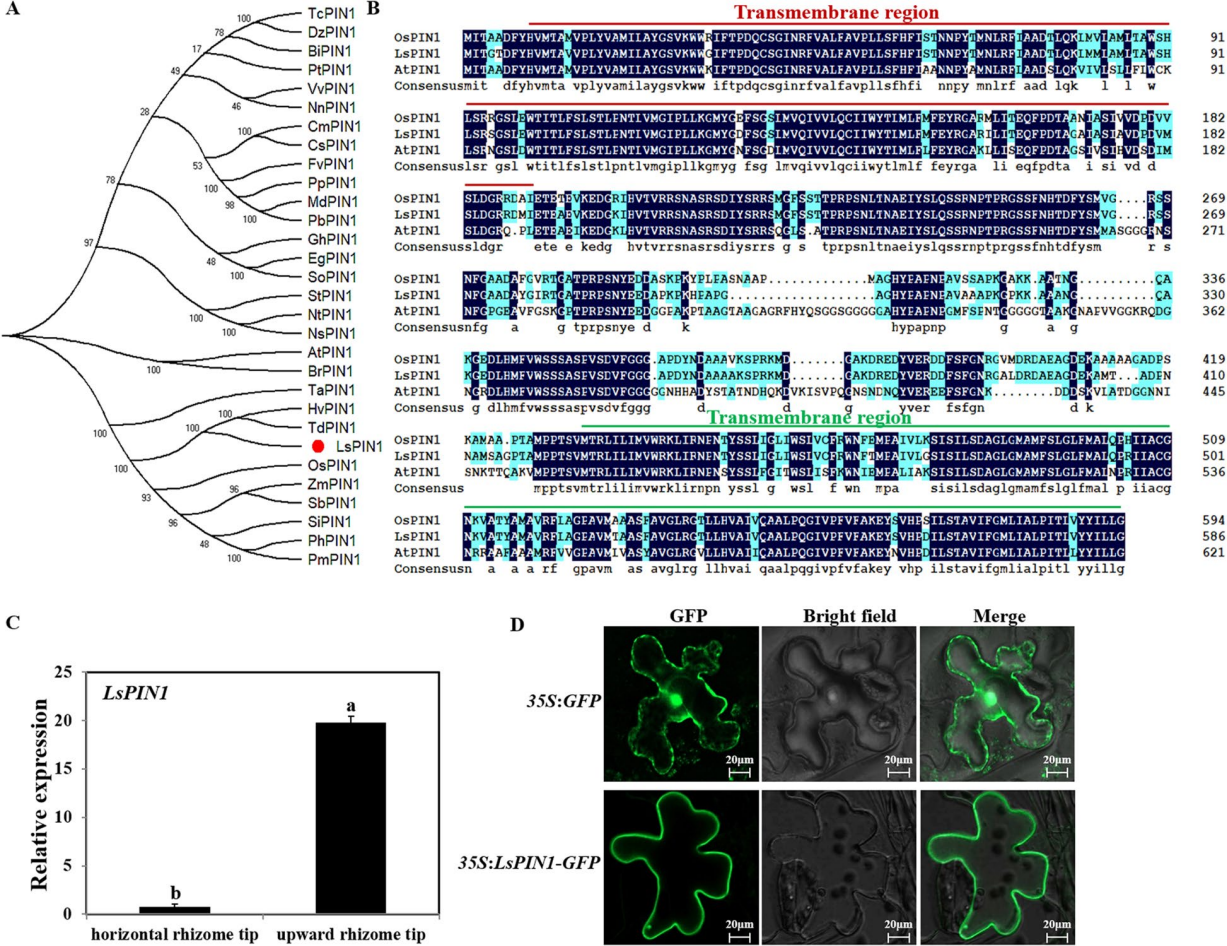
Multiple sequence alignment and phylogenetic analysis of the LsPIN1 protein of *L. secalinus* and PIN1 proteins from other species, and subcellular localization of LsPIN1 protein in tobacco leaf cells. **a** The full-length amino acid sequences of 30 PINs were used to build the phylogenetic tree with MEGA 7.0 software. The LsPIN1 was marked with the red circle. **b** Multiple sequence alignment of LsPIN1, OsPIN1, and AtPIN1 was conducted using ClustalX 1.81 with default parameters. **c** Transcript levels of *LsPIN1* in horizontal rhizome tips and upward rhizome tips. Error bars were the standard errors (SE). Different lowercase letters represented significant differences (*P* < 0.05). **d** Transient expression of *35S*:*LsPIN1*-*GFP* and *35S*:*GFP* in tobacco leaf cells. After 48 h of transformation, a confocal microscope was used to observe the green fluorescence signal. Bars = 20 μm

Transcriptome sequencing was conducted on the horizontal and upward rhizome tips in a previous study to clarify differences in gene expression between horizontal and upward rhizome tips. Several differentially expressed genes involved in auxin transport were identified in this study. One of these encoded a protein that was highly similar to the AtPIN1 protein; this gene, which we named *LsPIN1*, contained a 1755-bp coding sequence (CDS) (Fig. S1). Multiple-sequence alignment analyses of LsPIN1 and PIN1 protein sequences from rice and *A. thaliana* revealed that the transmembrane region and characteristic sequences of the *PIN* gene family were highly conserved (Fig. 4b). We constructed a phylogenetic tree using the neighbor-joining (NJ) method to clarify evolutionary relationships among LsPIN1 and PIN1 from other plant species [36]. As shown in Fig. 4a, the predicted LsPIN1 protein was nested within the same clade containing OsPIN1, which has been shown to play key roles in root formation and tillering [31]. LsPIN1 showed the highest homology with TdPIN1 and HvPIN1 from *Triticum dicoccoides* and *Hordeum vulgare*, respectively. The expression levels of *LsPIN1* in upward rhizome tips was higher than that in horizontal rhizome tips, and this was consistent with the transcriptome data (Fig. 4c). Sequence alignment and evolutionary analyses revealed that *LsPIN1* might encode an auxin transporter involved in rhizome development.

The subcellular localization of LsPIN1 was determined by introducing the fusion protein vector *35S*:*LsPIN1*-*GFP* into epidermal cells from tobacco leaves. The transgenic tobacco leaves carrying the *35S*:*GFP* empty vector were used as the negative control. The fluorescence signal corresponding to the *35S*:*LsPIN1*-*GFP* fusion protein was only observed in the membrane. The *35S*:*GFP* fluorescence was observed throughout the whole cell in the control (Fig. 4d), indicating that the LsPIN1 protein was a membrane localization protein, which was consistent with the previous prediction (Fig. S2).

### Heterologous over-expression of *LsPIN1* in *Arabidopsis* affects root development

**Fig. 5:**
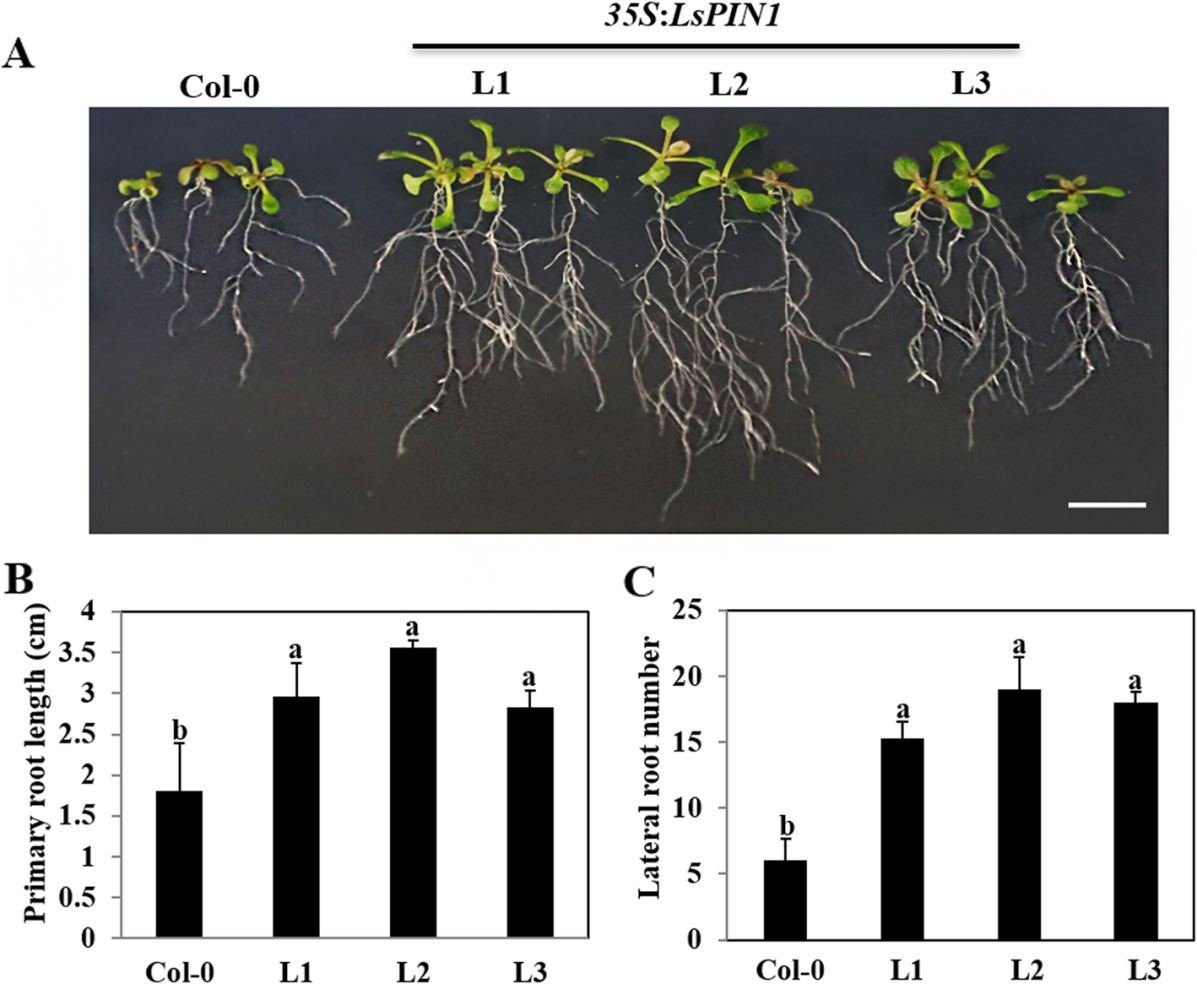
Overexpression of *LsPIN1* affected root development in *A. thaliana*. **a** Phenotypes of Col-0 and *LsPIN1*-overexpressing seedlings. **b** Length of the primary roots of 14-d-old *35S*:*LsPIN1* transgenic seedlings (L1, L2, and L3) and Col-0 seedlings. **c** Lateral root number in (**b**). (Bar = 1 cm). All experiments were conducted three to four times, and the results of each replication were similar. Error bars indicated SE calculated from the results of three independent experiments. Different lowercase letters represented significant differences (*P* < 0.05)

Given the difficulty of obtaining transgenic *L. secalinus* seedlings overexpressing *LsPIN1*, we used *Arabidopsis* seedlings for genetic transformation to determine the function of *LsPIN1*. Three independent transgenic lines (L1, L2, and L3) were used in subsequent analyses (Fig. 5, Fig. S3). The *35S*:*LsPIN1* transgenic *A. thaliana* and wild-type (Col-0) seedlings were grown on MS medium for approximately 1 week, then seedlings were transferred to squares (13 cm × 13 cm) with MS medium for vertical culture for 7 d. The *LsPIN1*-overexpressing seedlings grew larger overall and had significantly larger leaves than Col-0 seedlings. The primary roots (PRs) were significantly longer and the number of LRs significantly higher in *LsPIN1*-overexpressing seedlings than in Col-0 seedlings; specifically, the PRs were approximately 20% longer and the number of LRs approximately 40% greater in *LsPIN1*-overexpressing seedlings than in Col-0 seedlings (Fig. 5). These findings indicated that *LsPIN1* plays a role in regulating the growth and development of PRs, as well as LR morphogenesis.

### *LsPIN1* mediates auxin transport by responding to gravity signals

The distribution of auxin in various tissues plays a key role in regulating plant growth and development [37, 38]. As is well-known that auxin is essential for root development, and PIN1 has been shown to mediate the transport of auxin [30, 36, 39, 40]. Geotropic growth is a key feature of plant root morphogenesis, and auxin is the main signal mediating the response to gravity stimulation in plants. To determine whether *LsPIN1* affects root development by regulating auxin transport, gravitropic response and phototropic response assays were conducted. In the gravitropic response assays, plants were subjected to gravity and microgravity conditions.

**Fig. 6:**
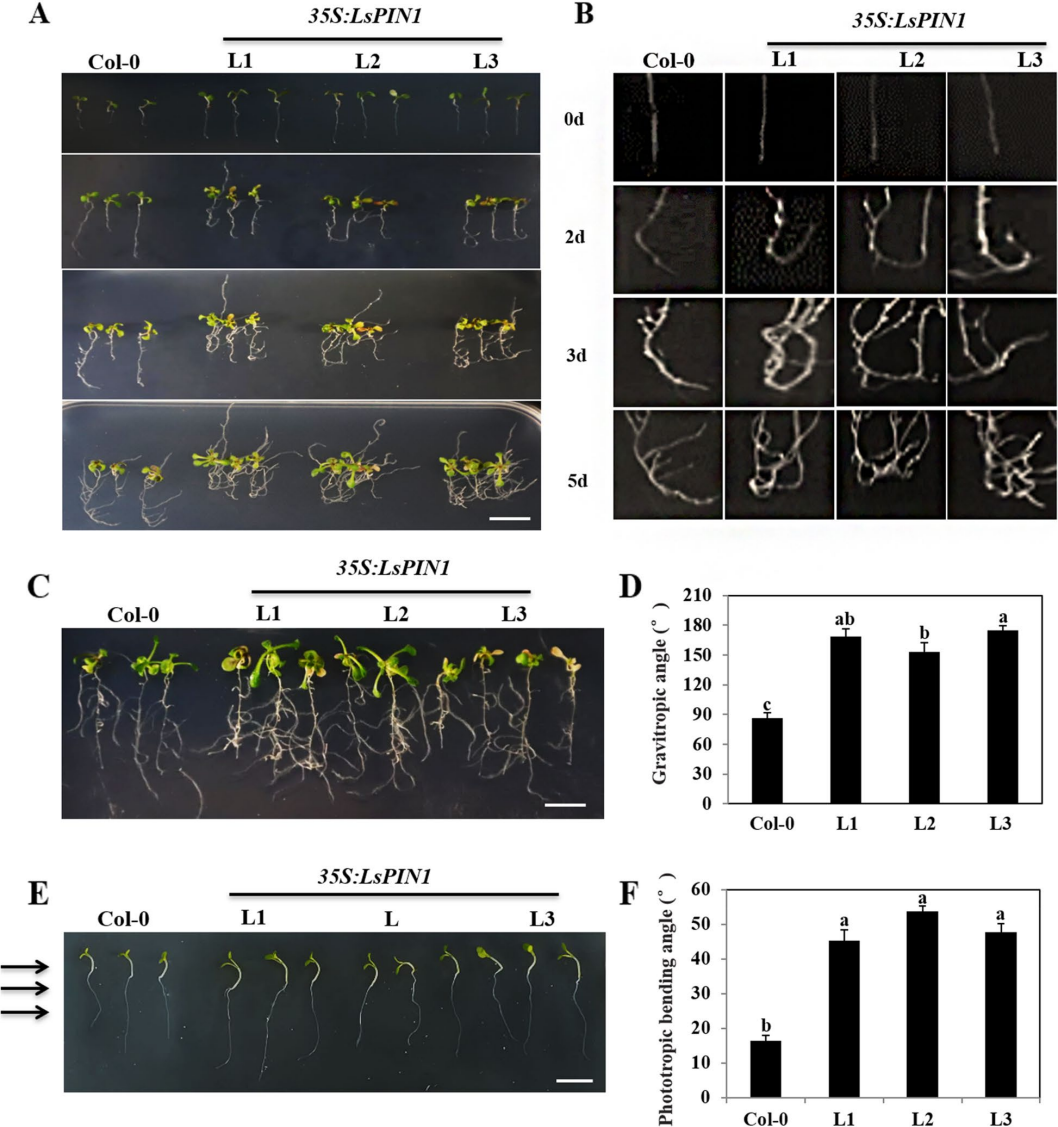
Gravitropic and phototropic responses in *LsPIN1*-overexpressing seedlings. **a** Phenotypes of *LsPIN1*-overexpressing seedlings and Col-0 seedlings were characterized at 2, 3, and 5 d following gravistimulation by rotating their culture dishes 135° from the vertical. **b** The turning angle of the root tips in (**a**). **c** Phenotypes of Col-0 and *LsPIN1* transgenic seedlings (L1, L2, and L3) after 7 d under simulated microgravity conditions on a monoaxial clinostat. **d** The gravitropic angle was measured for 10 roots per line using ImageJ software. **e** The phenotypes of transgenic and Col-0 seedlings were characterized after being placed in a dark environment with 12 h of unilateral light stimulation. The arrow indicated the orientation of light. Scale bars: 1 cm. **f** The bending angle of hypocotyl was measured by ImageJ software. Average bending angle measurements were taken from 20 seedlings. All experiments were repeated three to four times, and the results of each replication were similar. Error bars indicated SE calculated from the results of three independent experiments. Different lowercase letters represented significant differences (*P* < 0.05)

Under gravity conditions, Col-0 and *35S*:*LsPIN1* transgenic seedlings growing in the same MS medium were gravistimulated by rotating their culture dishes 135° from the vertical. After 0, 2, 3 and 5 d of continuous stimulation, the PRs and LRs of transgenic seedlings were more curved than those of Col-0 (Fig. 6a-b). The response of *LsPIN1* transgenic seedlings to gravity stimulation was stronger than that of Col-0, and the root turning angle was greater in the former than in the latter, suggesting that *LsPIN1* overexpression promoted geotropism during root growth (Fig. 6b). Under microgravity conditions, the growth rate and bending angle of the adventitious roots were significantly higher in various directions of *35S*:*LsPIN1* seedlings (L1, L2, and L3) than in Col-0 and *35S*:*LsPIN1* seedlings under normal conditions (Fig. 6c-d). Light signal is a key environmental factor affecting plant growth and development, and it has been proved to regulate auxin transport [41, 42]. In the phototropic experiment, the phototropic bending of the hypocotyl was more pronounced in transgenic *A. thaliana* seedlings than in Col-0 seedlings, and the bending angle of the hypocotyl was significantly higher in *A. thaliana* transgenic seedlings than in Col-0 seedlings (Fig. 6e-f). In sum, *LsPIN1* affected root growth and bending via the gravitropic response and phototropism.

### Overexpression of *LsPIN1* affects the accumulation of auxin

**Fig. 7:**
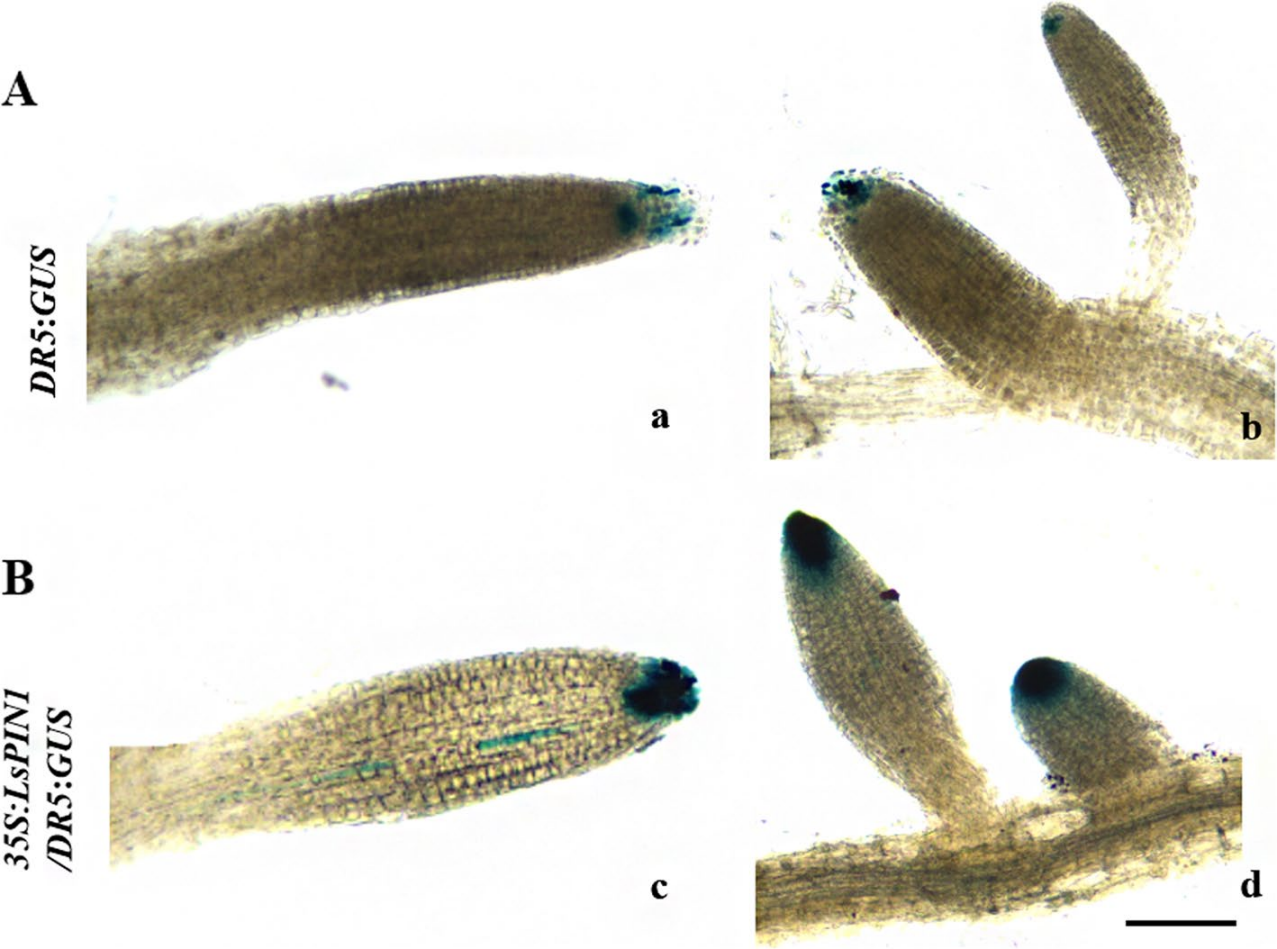
Patterns of auxin accumulation in *LsPIN1*-overexpressing seedlings. **a** Auxin accumulation in the primary root tips (a) and lateral root tips (b) of control seedlings (*DR5*:*GUS*). **b** Auxin accumulation in the primary root tips (c) and lateral root tips (d) of *35S*:*LsPIN1* transgenic seedlings with a *DR5*:*GUS* genetic background. Scale bars: 0.1 mm

Overexpression of *LsPIN1* affected root development and auxin transport, suggesting that it might also affect auxin accumulation in roots. The *35S*:*LsPIN1* overexpression vector was transferred into *A. thaliana* transgenic seedlings with the auxin responsive reporter gene *DR5*:*GUS*. *GUS* activity was significantly higher in the PR and LR tips of *LsPIN1*-overexpressing seedlings than in *DR5*:*GUS* control seedlings, indicating that the heterologous expression of *LsPIN1* affected the accumulation of auxin in *A. thaliana* (Fig. 7).

## Discussion

The potential applications of rhizomatous and clonal species of the genus *Leymus* are broad. First, *Leymus* species are widespread and excellent forages and show prolific clonal rhizomatous growth. Second, rhizomatous grasses are highly resistant to drought, saline-alkaline, and low-temperature stress; they can also withstand heavy trampling and various pests and diseases, which makes them suitable for ecological restoration [32]. Third, these grasses are rich in stress resistance genes, and some of these genes can be used to enhance the properties of cereal crops. Thus, studies of rhizomatous grasses can provide key information that could aid the recovery of degraded, desertified grassland vegetation and enhance the tillering traits of cereal crops. Such studies could also provide new insights that could aid the development of methods to impede the growth of harmful grasses and invasive species.

In recent years, many important advances have been made in the understanding of the regulatory mechanisms shaping tiller phenotypes and initial rhizome orientation in major gramineous crops, such as rice and barley [43–45]. In barley, *the als* recessive mutant had fewer LRs with few tillers and showed irregular inflorescence development, and *the Lnt1* recessive mutant had no secondary tillers and only one to four tillers at maturity [43, 44]. A total of 48 transcription factors, including transcription factors from the *AP2*, *bHLH*, and *NAM* families, were specifically expressed or highly enriched in the rhizome tips and elongation zones of rice [45]. OsTB1, as a negative regulator, was involved in the tillering process of rice [25]. The *LAX* (lax panicle) and *SPA* (small panicle) genes were major regulators involved in axillary meristem formation in rice; *LAX1* was involved in all types of axillary meristem formation during the whole growth cycle of rice [46, 47]. *DWARF* genes had also been shown to be involved in the growth of tiller buds [48]. The gradient distribution of plant hormones and the ratio of different hormones superimposed with the growth and development of plants together form a very sophisticated regulatory network. Several hormone-related genes that play a role in rhizome development had been identified, such as *EIN1* (ethylene insensitive 1), *AHK3* (auxin *Arabidopsis* histidine kinase 3), *MdPIN1*, and *AtPIN1* [3, 7, 30, 49]. The *PIN1* family gene *OsPIN1* was involved in adventitious root budding and tillering in rice, both of which were dependent on auxin [31]. The formation of rice tillering was affected by the overexpression of *OsIAGLU*, which encoded an IAA-binding enzyme, and exogenous IAA treatment [50]. The expression of auxin response factor 8 and auxin efflux vector 3 was down-regulated in the rhizome tips of *O. longistaminata* [51]. Although the functions and molecular mechanisms of some genes and hormones in root growth and tillering development have been studied, the genetic analysis, gene mapping, cloning and molecular regulatory mechanism of tillering characters of gramineous herbage, which are often used as important gene pool resources for cereal crop breeding, are still lagging behind.

Identification of the genes and factors that play key roles in the formation of horizontal and upward rhizome tips can enhance our understanding of the differentiation of rhizomes into different tissues and facilitate rapid canopy establishment. Previous studies have shown that plant hormones play major roles in the growth and development of roots and rhizomes. We first compared the content of endogenous hormones between horizontal and upward rhizome tips of *L. secalinus*. The content of GA_3_ and IAA was higher in upward rhizome tips than in horizontal rhizome tips, and the content of Me-JA and BR was higher in horizontal rhizome tips than in upward rhizome tips, suggesting that these hormones might mediate rhizome turning (Fig. 1e-h−. The number of rhizomes per plant and the ratio of rhizome turning number to total rhizome number were higher when *L. secalinus* seedlings were cultured with a hydroponic solution containing appropriate IAA and GA_3_ compared with seedlings that were cultured with the same solution without these hormones (Fig. 2, Fig. 3).

PINs are involved in several aspects of plant growth and development by regulating the transport and distribution of auxin. Eight *PIN* genes (*AtPIN1*-*AtPIN8*) have been identified in *A. thaliana*, and all of the proteins encoded by these genes are localized to the cell membrane. It is because of the existence of these carriers that it is effective for the auxin transport and plant growth. Several studies have shown that *AtPIN1*, which was the first *PIN* gene to be identified, plays a key role in regulating auxin transport. According to the transcriptome data measured in the early stage, we screened an auxin efflux carrier named LsPIN1. LsPIN1 was a membrane localization protein that was highly similar to the protein sequences of AtPIN1 and OsPIN1, and it had the highest homology with TdPIN1 and HvPIN1 from *T. dicoccoides* and *H. vulgare* according to evolutionary analysis, respectively (Fig. 4a-d). We hypothesized that LsPIN1 in *L. secalinus* might be an important transporter affecting auxin transport as well as root and rhizome development, similar to AtPIN1 in *A. thaliana*. Thus, we evaluated whether LsPIN1 regulated root development by overexpressing *LsPIN1* in *A. thaliana*. The length of the PRs was approximately 20% higher and the number of LRs was approximately 40% higher in *LsPIN1*-overexpressing plants than in control plants (Fig. 5). In the gravitropic response and phototropic assays, *LsPIN1* overexpression affected auxin transport and root bending, as *LsPIN1*-overexpressing plants showed stronger gravitropic and phototropic responses (Fig. 6). Meanwhile, the *DR5*:*GUS* reporter vector assay revealed that *LsPIN1*-overexpressing seedlings accumulated more auxin in both the PRs and LRs compared with control plants (Fig. 7). The functions of *LsPIN1* in *A. thaliana* were examined, and we found that LsPIN1 was involved in the growth and development of roots and auxin transport. The role of LsPIN1 in rhizome formation and turning in *L. secalinus* was not analyzed. It is known that the root of *Arabidopsis* could not upward grow and form tiller buds, so it is not a suitable material to study the function of *LsPIN1* in rhizome development and turning of *Leymus secalinus*. However, genes are often functionally conserved in different species. Studying the function of *LsPIN1* in the model plant *Arabidopsis* can be used as a reference to preliminarily reflect its possible role in rhizome development or turning of *L. secalinus*, so these findings provide new insights that will aid future studies examining the function of *LsPIN1* in *L. secalinus*.

## Conclusions

In this study, we detected the content of endogenous hormones in the horizontal rhizome tips and upward rhizome tips of *L. secalinus*. We found that the content of GA_3_ and IAA significantly differed between horizontal and upward rhizome tips. *LsPIN1* overexpression in *A. thaliana* increased the length of PRs and the number of LRs. The gravitropic response and phototropic response assays indicated that *LsPIN1* affected auxin transport. *LsPIN1* also promoted the accumulation of auxin in root tips. These findings indicated that *LsPIN1* might be involved in rhizome development in *L. secalinus*. This gene could thus potentially be used to ameliorate root and rhizome development in *L. secalinus* and other plants.

## Methods

### Plant materials and growth conditions

*L. secalinus* seeds were provided by Professor Jin Yi of the Forage Research Office of Inner Mongolia Agricultural University. These seeds were soaked in running water for 3-5 h, evenly spread on Petri dishes with moist filter paper, and incubated in an incubator at 28 °C for 5-7 d until the seeds germinated. The *L. secalinus* seedlings were grown in a greenhouse with a regime of 16 h light/8 h dark and at 28 °C. Seedlings with well growth were selected and transplanted into nutrient sandy soil or plastic buckets filled with an inflatable nutrient solution for root sampling and hormone treatment experiments. Gene cloning and determination of plant hormones were conducted using the rhizomes of *L. secalinus*. *Arabidopsis thaliana* (Columbia: Col-0), preserved in our laboratory, was used for genetic transformation. The *Arabidopsis* seedlings were grown in incubators at a constant temperature of 22 °C and under a 16-h/8-h day/night photoperiod.

### Quantification of plant hormones in horizontal and upward rhizome tips

Two-cm lengths of horizontal and upward rhizome tips were sampled from *L. secalinus* in the same growing environment, respectively; these samples were then immediately frozen in liquid nitrogen and stored at − 80 °C until they were used in subsequent experiments. A total of six separate rhizome tips were included in each sample, and there were 3-4 replicates for each sample. The content of ZR, ABA, GA_3_, Me-JA, IAA, and BR in horizontal and upward rhizome tips was measured using enzyme-linked immunosorbent assays (ELISAs). ELISAs were conducted following the manufacturer’s instructions as well as the procedures described in a previous study [52].

### Treatment of *L. secalinus* with exogenous hormones GA_3_ and IAA

To characterize the effects of different concentrations of GA_3_ and IAA on the rhizomes of *L. secalinus*, seedlings with similar growth status were transferred to 1-L nutrient solution for hydroponic growth. The seedlings were cultured in a hydroponic system with Hoagland nutrient solution for 2 d prior to adding hormones. Next, GA_3_ and IAA were added at various concentrations (GA_3_: 0 mg/mL (CK), 0.02 mg/L, 0.1 mg/L, 0.2 mg/L, 0.3 mg/L, and 0.4 mg/L; IAA: 0.005 mg/L, 0.01 mg/L, 0.02 mg/L, 0.04 mg/L, 0.05 mg/L, and 0.1 mg/L). The overall growth of seedlings and morphological changes in the rhizome under different GA_3_ and IAA concentrations were characterized at various time points.

### Multiple sequence alignment and phylogenetic analysis

Multiple sequence alignment of the LsPIN1, OsPIN1, and AtPIN1 proteins was conducted using ClustalX 1.81 with default parameters, and the sequence composition was determined using DNAMAN software (http://dnaman.software.informer.com/) [53]. The full-length amino acid sequences of 30 PIN1s were used to build an unrooted phylogenetic tree with MEGA 7.0 (https://www.megasoftware.net/), and the specific parameters used were based on those in previous studies [53]. The accession numbers and species corresponding to each of the PIN1 proteins were as follows: VvPIN1, XP_002282220.1 (*Vitis vinifera*); NnPIN1, XP_010263760.1 (*Nelumbo nucifera*); MdPIN1, MDP0000138035 (*Malus domestica*); GhPIN1, AMD39987.1 (*Gossypium hirsutum*); EgPIN1, XP_010036951.1 (*Eucalyptus grandis*); StPIN1, XP_006341527.1 (*Solanum tuberosum*); NtPIN1, XP_016514062.1 (*Nicotiana tabacum*); OsPIN1, NP_001388905.1 (*Oryza sativa*); AtPIN1, At1G73590.1 (*A. thaliana*); TcPIN1, XP_007036846.1 (*Theobroma cacao*); CmPIN1, XP_008464398.1 (*Cucumis melo*); BrPIN1, XP_009128000.1 (*Brassica rapa*); DzPIN1, XP_022775058.1 (*Durio zibethinus*); SoPIN1, XP_030460033.1 (*Syzygium oleosum*); CsPIN1, XP_011660216.1 (*Cucumis sativus*); PtPIN1, AAG17172.1 (*Populus tremula*); NsPIN1, XP_009783366.1 (*Nicotiana sylvestris*); PbPIN1, XP_009356039.1 (*Pyrus bretschneideri*); PpPIN1, XP_007210282.1 (*Prunus persica*); FvPIN1, XP_004299530.1 (*Fragaria vesca*); DzPIN1, XP_BlPIN1 (*Durio zibethinus*); BlPIN1, Alw04421.1 (*Betula luminifera*); HvPIN1, KAE8779542.1 (*H. vulgare*); ZmPIN1, XP_ 008646250.1 (*Zea mays*); PhPIN1, XP_025827259.1 (*Panicum hallii*); TdPIN1, XP_037451926.1 (*T. dicoccoides*); PmPIN1, RLM78735.1 (*Panicum miliaceum*); SiPIN1, XP_004953880.1 (*Setaria italica*); SbPIN1, XP_021315719.1 (*Sorghum bicolor*); and TaPIN1, AAS19858.1 (*Triticum aestivum*).

### Recombinant vector construction and *Arabidopsis* transformation

The complete coding sequence of *LsPIN1* was amplified and cloned into the pCAMBIA1300 vector with a GFP tag to yield *35S*:*LsPIN1*-GFP. *Agrobacterium tumefaciens* LBA4404 containing the recombinant vector was transformed into *A. thaliana* (Col-0) using a previously described procedure [54]. Homozygous T3 transgenic *A. thaliana* lines used in subsequent experiments were identified via hygromycin screening. Table S1 showed the primers that were used.

### Subcellular localization of LsPIN1

The empty *35S*:*GFP* vector and the recombinant *35S*:*LsPIN1*-*GFP* plasmid were inoculated into tobacco leaf epidermal cells to determine the subcellular localization of LsPIN1. Inoculated tobacco plants were grown under normal conditions for approximately 48 h, and the fluorescence signal was observed under a confocal laser scanning microscope LSM 800 (Zeiss).

### Gravitropic response assays and phototropic assays

Five-d-old wild-type and *35S*:*LsPIN1* transgenic *A. thaliana* were grown under normal conditions on MS Petri dishes. The roots were gravistimulated by rotating their dishes 135° from the vertical [30]. The root tip turning angle was measured after 2, 3, and 5 d of growth. Wild-type and *LsPIN1* transgenic lines were subjected to microgravity conditions by placing them on a monoaxial clinostat; the plants were cultivated at a constant temperature of 22 °C under a 16-h/8-h day/night photoperiod. The bending angle of 10 roots per line was measured using ImageJ software. Three biological replicates were conducted for both the control and treatments.

Five-d-old wild-type and *35S*:*LsPIN1* transgenic lines were placed in a dark environment for 3 d and cultivated under a unilateral light stimulus for 12 h to determine whether hypocotyls would bend towards the light [30]. The light intensity was measured using a digital LUX meter (TES-1332A, China). The light intensity of the light stimulus in the phototropic bending experiment was 4500 lx. Photographs of the plants at various time points were taken using a digital camera. ImageJ software was used to measure the bending angle of the hypocotyl. Average bending angle measurements from 20 seedlings were taken and used in subsequent analyses. Three biological replicates were conducted for both the control and treatments.

### *GUS* histochemical staining assays

*A. thaliana* with *DR5*:*GUS* (control) and *35S*:*LsPIN1* transgenic lines in a *DR5*:*GUS* genetic background were cultured in MS Petri dishes for 2 weeks. After the PRs and LRs appeared, control and transgenic seedlings were immersed in acetone to fix the internal cells, and then placed into a centrifuge tube with *GUS* staining solution to extract vacuum. Following 12 h of dark staining at 37 °C, 70% ethanol was used to decolorize seedlings. After complete decolorization (approximately 7-10 d), a confocal laser scanning microscope LSM 800 (Zeiss) was used to determine the locations at which auxin accumulated in both control and transgenic *GUS*-marked plants.

### Statistical analysis

In this study, the error bars represented the standard error (SE) from at least three biological replicates. The analysis of statistical significance was performed with the student’s t-test at *P* < 0.05 as described [55].

## Supplementary Information

**Additional file 1:** **Fig. S1**. The 1755-bp CDS of *LsPIN1* gene. **Fig. S2**. Predicted localization of LsPIN1: Plasma Membrane. **Fig. S3**. Identification of the overexpression of *LsPIN1* positive lines.

**Additional file 2:** **Table S1.** The primers used in this study.

**Additional file 3.**

## Data Availability

The data that support the results are included within the article, its additional files and in SRA database of NCBI. The accession ID is PRJNA776697, with the following link: https://www.ncbi.nlm.nih.gov/sra/PRJNA776697. Other relevant materials are available from the corresponding authors on reasonable request.

## Abbreviations

PIN: PIN-FORMED
At: *Arabidopsis thaliana*
Ls: *Leymus secalinus*
qRT-PCR: Quantitative reverse transcription-PCR
CDS: Coding Sequence
IAA: Indole-3-acetic acid
ABA: Abscisic acid
GUS: β-glucuronidase
