# Supplementary material for: Identification of LsPIN1 gene and its potential functions in rhizome turning of Leymus secalinus
Source: BMC Genomics. 2022 Nov 16;23:753. doi: 10.1186/s12864-022-08979-7 (PMC9670609; doi:10.1186/s12864-022-08979-7)
Supplement: Supplementary file 1 — Additional file 1: Fig. S1. The 1755-bp CDS of LsPIN1 gene. Fig. S2. Predicted localization of LsPIN1: Plasma Membrane. Fig. S3. Identification of the overexpression of LsPIN1 positive lines. [file 12864_2022_8979_MOESM1_ESM.pdf]

ATGATCACGGGCACGGACTTCTACCACGTCATGACGGCGATGGTGCCGCTGTACGTGGCGATGATGCTCG
CGTACGGGTCCGTCAAGTGGTGGCGAATCTTCACGCCGGACCAGTGCTCCGGGATCAACCGCTTCGTCG
CGCTCTTCGCCGTGCCGCTGCTCTCCTTCCACTTCATCTCCACCAACAACCCCTACACCATGAACCTTCG
CTTCATCGCCGCCGACACGCTGCAGAAGCTCATCGTGCTCGCGCTCCTCACCCTTTGGAGCCACCTCTCC
CGCAACGGCTCCCTCGAGTGGACCATCACGCTCTTCTCCCTCTCCACGCTGCCCAACACCCTCGTCATGG
GCATCCCGCTCCTCAAGGGCATGTACGGCGACGAGTCCGGCAGCCTCATGGTGCAGATCGTCGTCCTGC
AGTGCATCATATGGTACACGCTCATGCTCTTCATGTTCGAGTACCGCGGCGCCAGGATCCTCATCACGGA
GCAGTTCCCCGACACCGCCGGCGCCATCGCCTCCATCGCCGTCGACCCGGACGTCATGTCGCTCGACGG
CAGGAGGGACATGATCGAGACGGAGGCGGAGGTCAAGGAGGACGGCAAGATTCACGTCACCGTGCGCC
GCTCCAACGCCTCCCGCTCCGACATCTACTCGCGCCGCTCCATGGGGTTCTCCAGCACCACGCCGCGCC
CCAGCAACCTCACCAACGCCGAGATCTACTCTTTGCAATCGTCGCGGAACCCCACGCCCCGGGGCTCCA
GCTTCAACCACACCGACTTCTACTCCATGGTGGGCCGCAGCTCCAACTTCGCCGCCGGGGACGCGTTCG
GCCCGGTGGTGCGCACCGGGGCCACGCCGCGCCCGTCCAACTACGAGGAGGACAAGGCCGGCAACAA
CAACAAGTACGGGCAGTACCCGGCGCCCAACCCCGCGATGGCGGCGCCGCAGAAGCCGGCCAAGAAGG
CGGCCAACGGGCAGGCCAAGGGCGAGGACGGCAAGGACCTGCACATGTTCGTGTGGAGCTCGAGCGC
GTCGCCCGTGTCCGACGTGTTCGGCAACGGCACCGAGGCGTACAACGATGCCGCCGCCAAGGACGTTC
GCGTGGCCGCCGCCTCGCCGCGCAAAGCGGATGGCGTGGAGCGTGACGAGTTCAGCTTCGGGAACAAG
GAGAGGGACGCGGAGGCCGGCGACGAGAAGGCCGCGGCGGAGCAGGGCACCGCGGGCCTGGTGGCG
GCGCCCACGGCGATGCCGCCGACCAGCGTGATGACGCGGCTCATCCTCATCATGGTGTGGCGCAAGCTC
ATCCGCAACCCCAACACCTACTCCAGCCTCATCGGCCTCATCTGGTCCCTCGTCTGCTTCCGGTGGAACT
TCGAGATGCCGGCGATCATCATGAAGTCCATCGCCATCCTGTCCGACGCCGGCCTCGGCATGGCCATGTT
CAGCCTCGGGCTGTTCATGGCGCTGCAGCCGCGGATCATCGCGTGCGGGAACAAGCGGGCGACGTTCGC
CATGGCCGTGCGGTTCCTGACGGGCCCGGCCGTCATGGCCGCCGCCTCCATCGCCGTCGGCCTCCGCGG
CACCCTCCTCCACATCGCCATCGTGCAGGCAGCGCTGCCCCAGGGCATTGTCCCCTTCGTCTTCGCCAAG
GAGTACAGCGTGCACCCCGACATCCTCAGCACGGCTGTCATCTTCGGCATGCTCATCGCGCTGCCCATCA
CGCTGGTCTACTACATCCTGCTCGGCCTGTGA

Fig. S1. The 1755-bp CDS of *LsPIN1* gene.

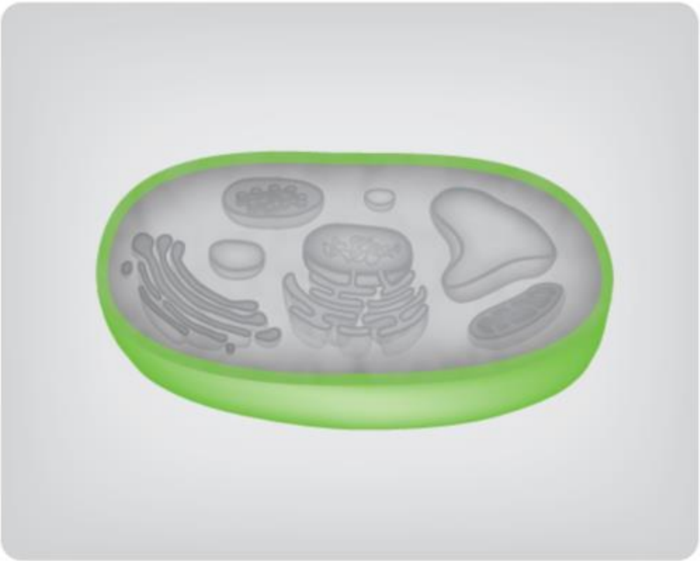

Fig. S2. Predicted localization of LsPIN1: Plasma Membrane.

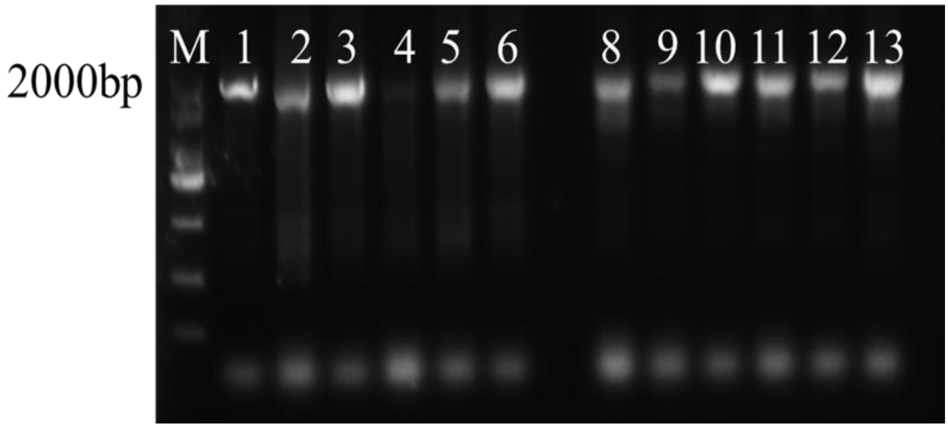

Fig. S3. Identification of the overexpression of *LsPIN1* positive lines.
