# Supplementary figures and images for: Identification of LsPIN1 gene and its potential functions in rhizome turning of Leymus secalinus
Source: BMC Genomics. 2022 Nov 16;23:753. doi: 10.1186/s12864-022-08979-7 (PMC9670609; doi:10.1186/s12864-022-08979-7)

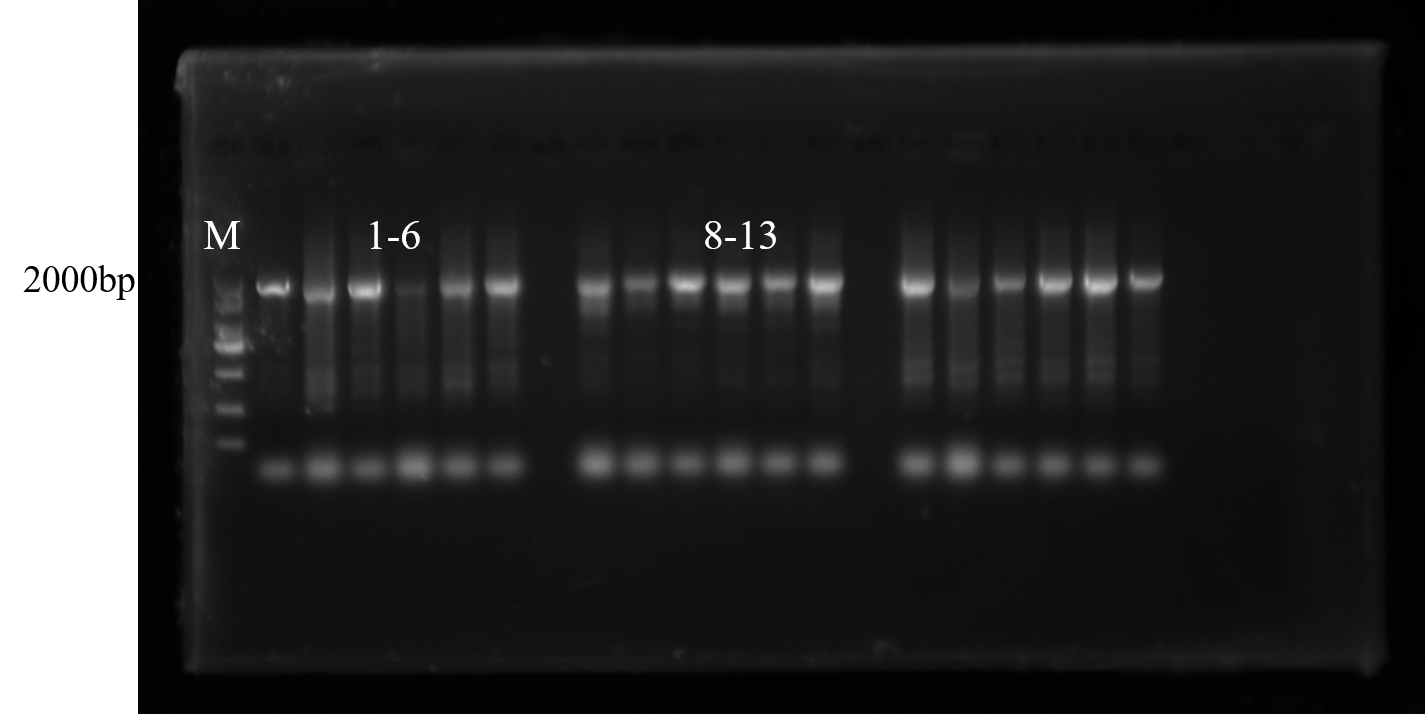

Supplement: Supplementary file 3 — Additional file 3. [file 12864_2022_8979_MOESM3_ESM.tif]
